# Differentiating features of OCT angiography in diabetic macular edema

**DOI:** 10.1038/s41598-021-02859-y

**Published:** 2021-12-03

**Authors:** Reza Mirshahi, Hamid Riazi-Esfahani, Elias Khalili Pour, Kaveh Fadakar, Parsa Yarmohamadi, Sayyed Amirpooya Alemzadeh, Samira Chaibakhsh, Khalil Ghasemi Falavarjani

**Affiliations:** 1 Eye Research Center, The Five Senses Health Institute, Rassoul Akram Hospital, Iran University of Medical Sciences, Tehran, Iran; 2 Eye Research Center, Farabi Eye Hospital, Tehran University of Medical Sciences, Tehran, Iran; 3 Young Researchers and Elite club, Tehran Medical Sciences, Islamic Azad University, Tehran, Iran; 4 Stem Cell and Regenerative Medicine Research Center, Iran University of Medical Sciences, Tehran, Iran

**Keywords:** Retinal diseases, Diabetes complications

## Abstract

The purpose of current study was to evaluate different optical coherence tomography angiography (OCTA) metrics in eyes with diabetic retinopathy with and without diabetic macular edema (DME). In this retrospective study, macular OCTA images of eyes with non-proliferative or proliferative diabetic retinopathy were evaluated. Vascular density, vascular complexity and non-perfusion densities were compared between eyes with and without DME. One-hundred-thirty-eight eyes of 92 diabetic patients including 49 eyes with DME were included. In multivariate analysis, the presence of DME was positively associated with geometric perfusion deficit (GPD) in superficial capillary plexus (SCP), capillary non-perfusion (CNP) of SCP, and GPD in deep capillary plexus (DCP) (all P < 0.05). In eyes with DME, central foveal thickness was associated with VD ratio (SCP/DCP) (P = 0.001) and FAZ area (P = 0.001). In conclusion, in eyes with diabetic retinopathy, the presence of DME was associated with more extensive capillary non-perfusion compared to those with no macular edema.

## Introduction

Diabetic retinopathy (DR) and diabetic macular edema (DME) are the main causes of severe visual loss in adult population^1,2^. Several studies have reported characteristic fluorescein angiography features associated with progression of diabetic retinopathy, and development and severity of diabetic macular edema^3–5^. Fluorescein angiography (FA), however, is an invasive and time-consuming modality that needs skilled photographers. Recently, optical coherence tomography angiography (OCTA), a non-invasive modality that provides depth-resolved images from retinal microvasculature has replaced FA in many patients^6,7^.

Different OCTA metrics have been shown to predict the presence and the stages of diabetic retinopathy^7–10^. However, studies reporting OCTA characteristics of diabetic eyes with macular edema are limited. Qualitative OCTA features have been described in eyes with DME^11–13^. In addition, OCTA changes have been reported after anti-VEGF or laser treatment^8,14,15^. However, research on the pattern of retinal ischemia in the setting of diabetic macular edema is limited.

The current study aims to assess different OCTA metrics in eyes with DME compared to diabetic eyes without macular edema.

## Methods

This retrospective case series was conducted in Rassoul Akram Hospital and Farabi Eye Hospital, Tehran, Iran. The study was approved by the Ethics Committee of Iran University of Medical Sciences (IR.IUMS.REC.1398.078) and adhered to the tenets of the Declaration of Helsinki. Informed consent was obtained from all participants.

Eyes with different stages of non-proliferative (NPDR) or proliferative diabetic retinopathy (PDR) were included. Patients underwent thorough ophthalmic examination including biomicroscopy and dilated indirect ophthalmoscopy. The Best Corrected visual acuity (BCVA) (using Snellen chart) was measured, and the results were then converted to logarithm of the minimum angle of resolution (LogMAR). Exclusion criteria were history of vitreoretinal surgery, history of macular photocoagulation, the presence of significant epiretinal membrane or vitreomacular traction and concurrent ocular diseases such as uveitis, glaucoma or optic neuropathy, eyes with visual acuity less than 20/200 and refractive error >  + 3 and <  − 3, and history of intravitreal injection of bevacizumab during the last 3 months.

### Imaging protocol

Optical coherence tomography angiography images were obtained from the fovea (3 × 3 mm) of the patients with different stage of diabetic retinopathy using a RTVue XR 100 Avanti instrument (Version 2017.1.0.151, Optovue, Inc., Fremont, CA, USA). Images with poor quality or from eyes with media opacity precluding acceptable image acquisition, were excluded from the study. Eyes with central subfield retinal thickness (CST) greater than 350 µm or presence of central retinal cystoid changes were considered as having diabetic macular edema.

Automated segmentation of the superficial capillary plexus (SCP) enface image was performed from internal limiting membrane (ILM) to 9 µm above the inner plexiform layer (IPL). Deep capillary plexus (DCP) boundaries were set at 9 µm above the IPL to 9 µm below the outer plexiform layer (OPL). Segmentation error correction of different retinal layers was manually performed as described elsewhere^16,17^. Foveal avascular zone was delineated in full retinal slab, SCP and DCP using a previously reported deep learning approach^18^.

For further analysis, SCP and DCP enface images were imported to into the ImageJ software (version 1.52; http://imagej.nih.gov/ij/; provided in the public domain by the National Institutes of Health, Bethesda, MD, USA). In order to reduce signal noise, Frangi vesselness filter was applied to the original image. Then, the images were binarized using the value of 1.2*SD + mean decorrelation signal of the deep learning delineated FAZ area as the threshold. The binary mask images were used for measuring different OCTA metrics as follows.

### Vascular density

Vascular density (VD) was defined as area of pixels occupied by retinal vessels divided by total area of the whole image.

### Vascular length density and vascular diameter index

Vascular length density (VLD) index was calculated as the area of pixels occupied with skeletonized vessels divided by the total area of the image. In skeletonization, reducing the width of each vessel to a single pixel results in uniform contribution of the large and small vasculature in density measurements^19^.

Vascular diameter index (VDI) was calculated by dividing VLD to VD. This index is a surrogate for total vessel length in the image.

### Complexity indices of retinal microvasculature

For measuring retinal vascular complexity, three different popular indices including fractal dimension (FD), vascular tortuosity index (VTI) and vascular complexity index (VCI) were calculated. Fractal dimension was calculated by FractalCount plugin of ImageJ software on skeletonized images of SCP and DCP using the box-counting method as described elsewhere^20^. FD shows the branching distribution of the vessels in the image^21^.

Vascular tortuosity index represents the curvature of retinal vasculature. It was calculated as the ratio of arch length (geodesic distance) to chord length (Euclidian distance) of each vessel segment in skeletonized image^22^.

Vascular complexity index was calculated based on binarized vessel map and vessel perimeter map. The complex morphology of retinal vasculature is measured by this unique index^22^. Generally, capillary drop out and non-perfusion results in less complex vascular network^23^.

$$\mathrm{It\, is\, calculated\, as}:\mathrm{ VCI}=\frac{{(\mathrm{Pixels\, enclosed\, by\, perimeter\, map})}^{2}}{4\pi \times (\mathrm{Pixels \,enclosed \,by\, vessel\, map})}$$

**Figure 1:**
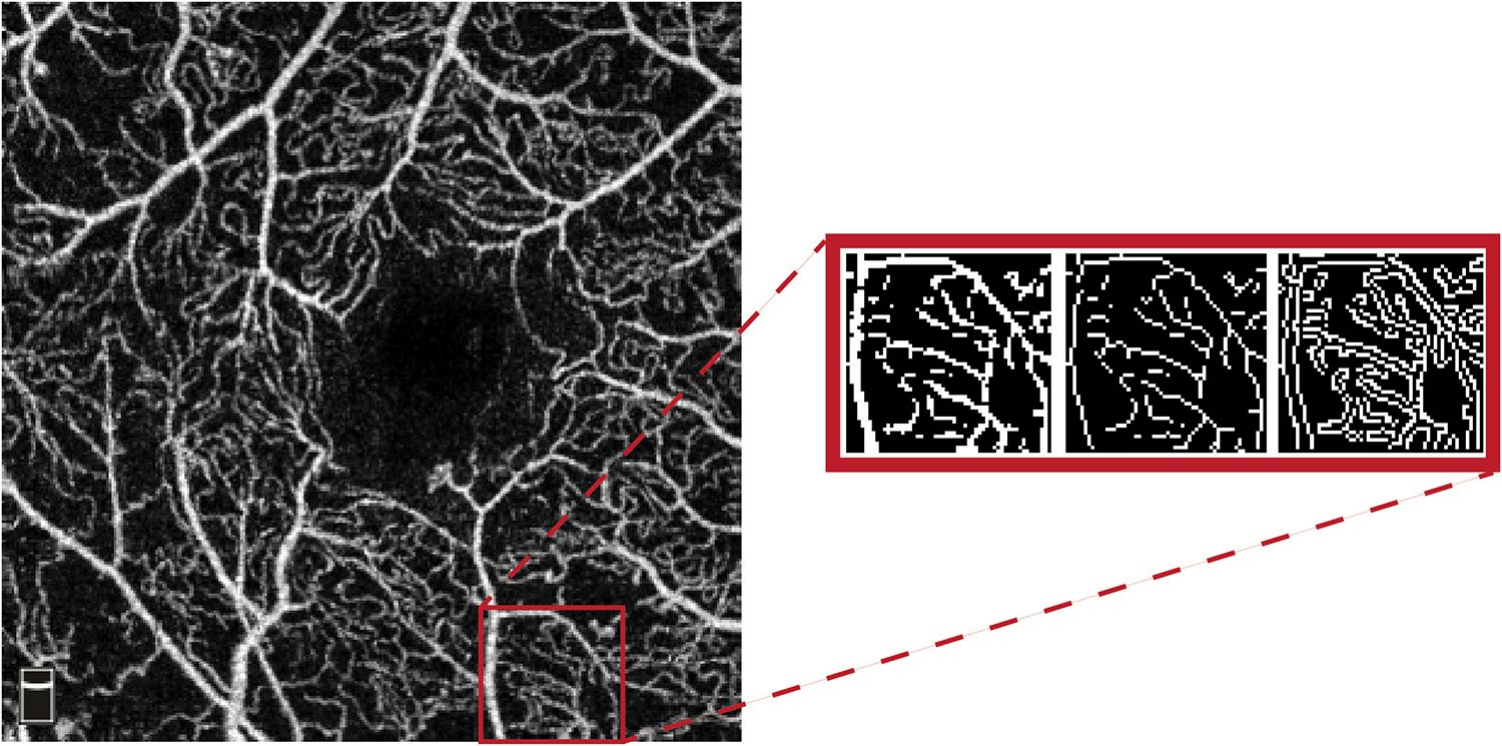
Optical coherence tomography angiography (OCTA) image of a patient with diabetic retinopathy (left image). The enlarged inset shows the enhanced binarized image, skeletonized image, and vessel perimeter map of a region of interest, from left to right, respectively. In the skeletonized image the vessels are thinned to a single pixel making the measurement of vessels’ length a simpler task in image processing. Vessel perimeter map outlines the perimeter of each vessel which is used for measuring the vascular complexity index. It should be noted that the region of interest shown in this image, was selected for better visualization of the image processing techniques. Whole image was used for all analyses.

The validity of all mentioned indices has been previously shown in different OCTA studies on diabetic patients^21–23^. Different obtained maps are illustrated in Fig. 1.

### Nonperfusion indices

**Figure 2:**
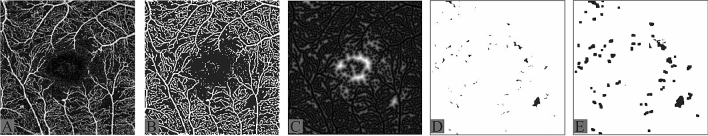
Original enface optical coherence tomography angiography (OCTA) image of a patient with diabetic retinopathy in superficial capillary plexus (SCP) layer **(A)**. The Frangi vesselness filter is applied and the image is binarized **(B)**. The vessel distance map **(C)** is generated to show the distance of each pixel from the nearest vessel. This map is then thresholded **(D)** to eliminate the areas related to normal inter-capillary distance. Finally, after applying morphological filters based on previous experience in a normal database, capillary nonperfusion **(E)** area (dark pixels: 4.07%) is calculated.

**Figure 3:**
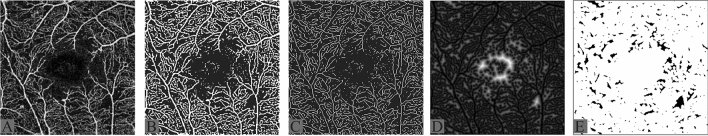
Original enface optical coherence tomography angiography (OCTA) image of a patient with diabetic retinopathy in superficial capillary plexus (SCP) layer **(A)**. The Frangi vesselness filter is applied and the image is binarized **(B)**. The image is then skeletonized **(C)** and the vessel distance map **(D)** is generated based on the skeletonized image to show the distance of each pixel from the nearest vessel. This map is then thresholded **(E)** to eliminate the areas related to normal inter-capillary distance, and geometric perfusion deficit area (dark pixels: 5.96%) is calculated.

Two different methods were implemented for quantification of capillary non perfusion (CNP). First, as described elsewhere^24^, quantification of CNP percentage in whole enface SCP and DCP images was done after enhancing images for generation of vessel distance map. Morphologic filters including Gray Scale Attribute Filtering and Erosion were applied afterwards for better visualization of CNP area (Fig. 2). Additionally, a similar method was used based on Euclidean distance map obtained from skeletonized image to calculated geometric perfusion deficit (GPD, Fig. 3)^25^. The FAZ area delineated by DL method was excluded from nonperfusion areas.

### Statistical analysis

Data entered using a SPSS software (SPSS, Inc, Chicago, IL). For comparing demographic variables of patients, between Macular edema and no Macular edema groups, independent t and chi-square tests were utilized. The generalized estimating equation (GEE) model was used for analysis of the extracted densities and indices to compensate for inter-eye correlation of bilateral cases. In statistical analysis, the results were adjusted for the stage of diabetic retinopathy (NPDR vs. PDR). P value less than 0.05 was considered significant. Bonferroni correction was used to address multiple comparisons.

## Results

One-hundred-thirty-eight eyes of 92 diabetic patients with a mean age of 62.2 ± 9.8 years were included. Fifty-nine eyes (42.7%) had diabetic macular edema. Forty-four eyes (31.9%) had PDR and the remaining eyes were categorized as NPDR. The mean CST was 420.1 ± 115.4 and 254.1 ± 29.9 µm in eyes with and without DME, respectively (P < 0.001). Table 1 shows demographics of patients in the 2 groups.

**Table.1:** Demographics of patients with diabetic retinopathy with and without diabetic macular edema.

Variable	Macular edema (59 eyes)	No macular edema (79 eyes)	P
Age	63.4 ± 10.2	60.6 ± 8.8	0.163*
Sex (female)	23 (50.0%)	30 (65.2%)	0.140^†^
Diabetic retinopathy (proliferative vs non-proliferative)	40/19	54/25	0.544^†^
Best corrected visual acuity (logMAR)	0.46 ± 0.30	0.19 ± 0.19	< 0.001*
Central subfield thickness (µm)	420.1 ± 115.4	254.1 ± 29.9	< 0.001*

*T test.

^†^Chi square test.

**Figure 4:**
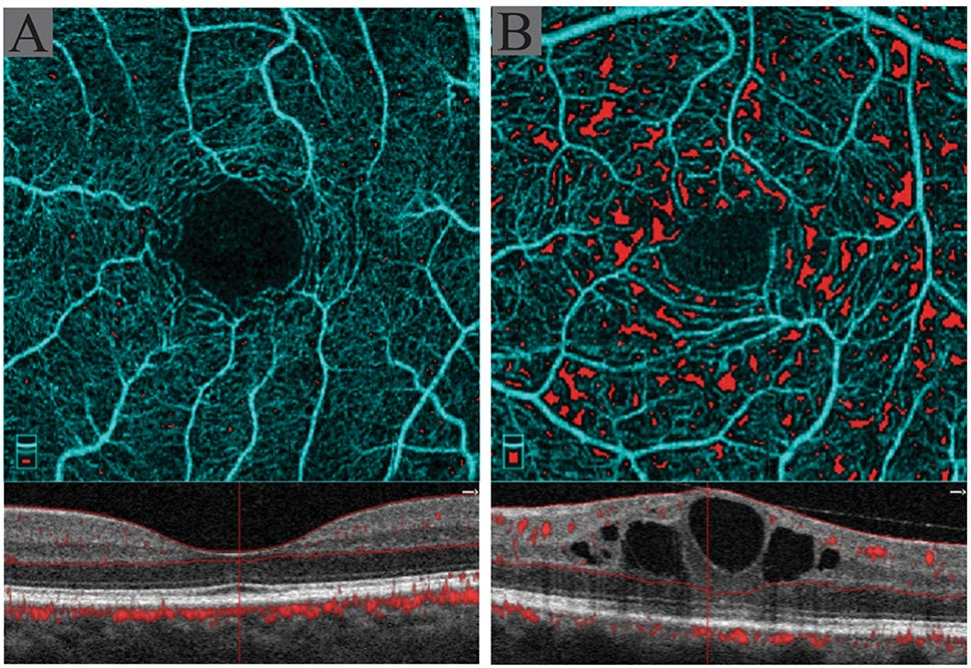
Enface optical coherence tomography angiography (OCTA) and the corresponding OCT B-scan image of two age-matched patients with similar stage of diabetic retinopathy showing lesser extent of capillary non-perfusion in patient without diabetic macular edema **(A)** in comparison to the patient with macular edema **(B)**. Red colors in enface images show geometric perfusion deficit (0.17% versus 5.67%).

Table 2 shows a comparison of different OCTA metrics in eyes with and without DME. In multivariate analysis, after adjusting for the stage of diabetic retinopathy and Bonferroni correction, the presence of diabetic macular edema was associated with higher GPD in SCP (p < 0.001), more CNP of SCP (p = 0.002), and higher GPD in DCP (p = 0.004). Figure 4 shows an example of enface OCTA images of patients with and without DME.

**Table.2:** Comparison of optical coherence tomography angiography (OCTA) metrics in eyes with and without diabetic macular edema based on univariate analysis.

Variable	Macular edema	No macular edema	P value*
FAZ (mm^2^)	0.46 ± 0.18	0.46 ± 0.16	0.925
VTI SCP	1.130 ± 0.016	1.138 ± 0.013	0.009
FD SCP	1.942 ± 0.019	1.944 ± 0.012	0.385
FD DCP	1.958 ± 0.009	1.965 ± 0.004	0.002
CNP SCP (%)	8.35 ± 6.37	4.99 ± 4.17	0.034
GPD SCP (%)	9.91 ± 5.22	7.01 ± 3.95	0.022
VD SCP (%)	26.37 ± 3.75	28.86 ± 3.77	0.004
VTI DCP	1.131 ± 0.016	1.141 ± 0.013	0.005
CNP DCP (%)	2.85 ± 2.85	1.09 ± 1.27	0.003
GPD DCP (%)	3.42 ± 2.21	2.17 ± 1.61	0.031
VD DCP (%)	27.61 ± 4.22	31.54 ± 3.63	0.002
VDI SCP	2.26 ± 0.10	2.31 ± 0.08	0.011
VDI DCP	2.22 ± 0.09	2.24 ± 0.07	0.149
VCI SCP	1.09 ± 0.08	1.09 ± 0.08	0.393
VCI DCP	1.20 ± 0.06	1.20 ± 0.05	0.835

*FAZ* foveal avascular zone, *VTI* vascular tortuosity index, *SCP* superficial capillary plexus, *DCP* deep capillary plexus, *FD* fractal dimension, *CNP* capillary non perfusion, *GPD* geometric perfusion deficit, *VD* vessel density, *VDI* vessel diameter index, *VCI* vascular complexity index.

*Based on GEE model after adjusting for the stage of diabetic retinopathy.

When calculating the ratio of non-perfusion area of SCP to DCP, both CNP ratio (5.65 ± 5.83 vs. 7.85 ± 6.96, p = 0.728) and GPD ratio (3.61 ± 1.80 vs. 4.20 ± 1.91, p = 0.417) were lower in eyes with diabetic macular edema. In addition, VD ratio of the SCP to DCP was higher in eyes with DME (0.96 ± 0.14 vs. 0.91 ± 0.07, P = 0.180).

In eyes with DME, central foveal thickness was only associated with increased VD ratio (p = 0.001) and larger FAZ area (p = 0.001). In eyes without DME, CST was only associated with FAZ area (p < 0.001) after Bonferroni correction.

## Discussion

In this study, the extent of capillary non-perfusion was higher and vessel density was lower in eyes with DME, in both SCP and DCP. We calculated the non-perfusion area based on 2 different methods to increase the accuracy of the measurements, and the results were similar^24,25^. Traditional staging of diabetic retinopathy based on Early Treatment of Diabetic Retinopathy Study (ETDRS), which is still the gold standard for its classification, describes the proliferative and non-proliferative stages of diabetic retinopathy^26^. The validity of this classification was further shown by evidence of increasing capillary non-perfusion with advancing ETDRS stage^27,28^. Diabetic macular edema was described in this classification as a condition that necessitates treatment with macular photocoagulation. With the advances in intravitreal pharmacological therapy, the indications, and the response of the treatment of DME have been changed. However, DME is not generally considered an ischemic process. Our results show that capillary non-perfusion and consequent ischemia are more severe in DME. It may be plausible to consider DME as a more advanced stage of diabetic retinopathy based on capillary non-perfusion.

In a previous study^29^, we showed that the extent of non-perfusion is higher in SCP than DCP in eyes with DR without DME. Similarly, current study shows that the non-perfusion area was more extensive in SCP. However, a lower (although non-statistically significant) capillary non-perfusion ratio (SCP to DCP) was observed in eyes with DME. In addition, severity of DME, evident as increase in CMT, was associated with a decrease in VD at DCP. This shows that in DME, the non-perfusion area in DCP is higher compared to those without macular edema. This is in line with previous studies that speculated a significant role for DCP ischemia in the pathogenesis of DME^11^.

Studies comparing the OCTA metrics between eyes with and without DME are scarce in the literature. In a prospective study by Sun et al.^13^ the development of DME was associated with decreased vascular density in SCP. AttaAllah et al.^30^ reported that eyes with DME had larger FAZ area and lower VD at DCP in comparison to eyes without DME. None of these studies reported the measurements of CNP or GPD area. Although the VD may indirectly indicate the perfusion status of the capillary plexus, the measurements of CNP and GPD directly show the status of perfusion. In our investigation, several additional image processing and deep learning techniques for measurement of OCTA metrics including VDI, FD, VTI, CNP and GPD were used to assess different aspects of non-perfusion in OCTA.

The segmentation error is one of the major sources of bias in quantitative measurements^16,31^ of OCTA images. In this study, the segmentation error was corrected manually in OCTA images. Despite this, some eyes with severe macular edema associated with disorganization of retinal layers may fail to be completely corrected. It might be speculated that the presence of cystoid spaces in different capillary plexuses might displace the vascular network laterally and affect the visualization of microvasculature. However, it is shown that reperfusion usually does not occur after the resolution of DME, therefore, to some extent the capillary drop out is authentic and not the result of artifacts associated with cystoid spaces^32^.

In this study, we analyzed the complexity indices of retinal microvasculature, including, FD, VTI and VCI. Previous studies have shown that vascular complexity indices are valuable measures in differentiating stages of DR^22,33^. Our results showed that VTI at SCP and DCP, VDI at DCP, and FD at SCP were different between DME and no DME groups. The difference, however, did not remain significant in multivariate analysis. Future studies with larger sample size may find an association between vascular characteristics and macular edema.

This study has several limitations. The study design was retrospective and sample size was not large enough to detect small differences between the groups. In addition, we did not evaluate the subcategories of NPDR, longitudinal changes in diabetic retinopathy and the changes after treatment in DME. Also, we did not assess structural changes in OCT b scans as it is shown that the inner retinal layers disorganization and paracentral acute middle maculopathy are related to macular ischemia in different levels of capillary plexuses^34^. Despite recent advances in OCTA software, many of artifacts cannot be readily fixed with present technologies^29^. Although low-quality images were excluded from this study and segmentation error was manually corrected, other forms of artifacts, especially due to cysts could have affected our measurements. Another drawback is the absence of baseline data regarding metabolic control among our patients. The intermediate capillary plexus (ICP) is also not discernible by the current Optovue device, and future studies can be conducted to assess the effect of DME on ICP.

In conclusion, this study evaluated a large set of OCTA variables and showed a possible role for non-perfusion in macular edema. Future longitudinal studies with larger sample sizes are needed to confirm our results.
